# Thermotherapy Effects on Healthy and Type 2 Diabetes Human Skeletal Muscle Myoblast Cell Lines

**DOI:** 10.1155/2021/9971599

**Published:** 2021-08-14

**Authors:** Janette A. Lindstrom, Felix Omoruyi, Jean Sparks

**Affiliations:** Department of Life Sciences, Texas A&M at Corpus Christi, Corpus Christi 78412, TX, USA

## Abstract

Diabetes mellitus is a chronic metabolic disease characterized by elevated blood glucose levels with associated disordered carbohydrate and lipid metabolism. Type 2 diabetes (T2D) specifically has been shown to cause a decrease in skeletal muscle mass due to oxidative stress. This study investigated a treatment option for T2D through thermotherapy on healthy (HSMM) and T2D (D-HSMM) human skeletal muscle cells. The goals were to determine the effects of thermotherapy, long-term (chronic) and short-term (acute), on HSMM and D-HSMM cell viabilities and oxidative stress. HSMM and D-HSMM cells were grown to confluency, harvested, and counted to determine density. Acute and chronic heat treatments were applied to both cell lines. The chronic treatment consisted of a 30-minute exposure to 40°C, three times a week for three weeks; the acute treatment was a one-time exposure. Oxidative stress assays and cell viabilities were tested 24 hours after heat treatments. Results indicated no significant effect on the cell viability of HSMM and D-HSMM cells. The acute treatment had a significant increase (*p* ≤ 0.05) of MDA concentration compared to the chronic treatment. The chronic treatment had a significant increase (*p* ≤ 0.05) in catalase activity compared to the acute treatment. The SOD activity had no significant change (*p* > 0.05) between the chronic and acute treatments. In conclusion, acute thermotherapy may not be beneficial for skeletal muscle cells due to the observed increase in oxidative stress, especially in the D-HSMM cells.

## 1. Introduction

Diabetes mellitus is a chronic metabolic disease characterized by elevated blood glucose levels with associated disordered carbohydrate and lipid metabolism. There are three main types of diabetes: type 1, type 2, and gestational. Type 1 diabetes is an autoimmune disorder of the T cells preventing the pancreatic production of insulin. The T cells destroy *β* cells in the pancreas which reduce insulin production. Type 2 diabetes (T2D) develops slowly with any cell type developing increasing resistance to insulin's action [1]. According to Chatterjee et al. [2], T2D is the most prevalent of the three forms. Approximately 400 million people worldwide in 2017 were diagnosed with T2D, and this is predicted to increase by an additional 200 million by 2040 [2]. Insulin resistance and biochemical tests help differentiate between type 1 and T2D. Gestational diabetes can occur during pregnancy due to hormonal changes in the mother that result in increased insulin resistance. It diminishes after the birth of the baby, and the hormones return to healthy levels. Women who experience gestational diabetes also have a predisposition to developing T2D [1]. As disordered carbohydrate and lipid metabolism are associated with diabetes, one component of the disorder that can be measured is oxidative stress.

Meng and Yu [3] defined oxidative stress as the “imbalance of oxidant and antioxidant levels.” Reactive oxidant species (ROS) can include superoxide anion (O_2_^−^), hydroxyl radical (OH), alkoxyl radical (RO), peroxyl radical (ROO), hydrogen peroxide (H_2_O_2_), and oxygen singlet (O_2_) [4]. Due to their highly reactive nature, an imbalance in favor of ROS in the human system causes deleterious effects as in the destruction of protein pathways related to age-associated muscle wasting [4, 5]. The mitochondria become dysfunctional in T2D patients due to the lack of glucose within the cell to produce energy, and the addition of ROS leads to apoptosis and cell death [3, 6]. T2D individuals experience increased levels of oxidative stress as a result of the ROS causing increased levels of non-metabolized lipids. These lipids can be measured as an oxidation reaction by-product, malondialdehyde (MDA) [3, 6–8]. Additionally, ROS promotes increased inflammation which causes an increase of cytokines-interleukins (IL) and tumor necrosis factor (TNF–*α*) [3, 4, 9]. Unchecked increased levels of ROS result in cell death. The human body has endogenous antioxidant systems to hinder this process by increasing the levels of superoxide dismutase (SOD) and catalase [4].

Three common markers used to assess oxidative stress include: MDA, SOD, and catalase activity [7, 10–15]. Lipid peroxidation of cells results in an increased reactive compound MDA production [7, 8]. The MDA is produced due to the presence of unsaturated fatty acids in the cellular membrane. The ROS reacts with the unsaturated fatty acids' bis-allylic methylene groups (low bond-dissociation energies of the methylene carbon-hydrogen bonds) to form more reactive products such as conjugated dienes, lipid hydroperoxides, F2-isoprostane, and MDA [8, 16, 17]. The ROS reactivity with the unsaturated fatty acids damages the cell membrane's fluidity and permeability. The reactive by-products can interact and damage proteins and DNA to cause further cellular damage [17–19]. SOD and catalase are markers of the cell's defense against ROS as these markers prevent and restore cellular damage and functional impairments via nullification of the reactive radicals [4, 17, 20–22]. SOD activity produces H_2_O_2_ molecules from the ROS′ superoxide anions (O_2_^−^). The H_2_O_2_ molecules are less reactive compared to other groups of ROS [17]. The catalase completes the ROS removal process through catalyzing H_2_O_2_ molecules into H_2_O and O_2_ molecules (and other free radicals into neutral molecules) that the SOD began [22]. SOD and catalase work together to protect against oxidative cellular damage.

The T2D skeletal muscle cells' inability to intake glucose combined with increased oxidative stress have been shown to decrease muscle mass, a characteristic of sarcopenia [3, 6, 23]. Within the human body, skeletal muscle cells make up to 80% of all cell types in relation to the action of the insulin-sensitive glucose metabolism [24]. Skeletal muscle cells differ from other cell types by containing muscle-specific transcriptional factors such as MyoD and Myf5 [25, 26]. They originate from myoblasts and then become myogenic precursor cells producing the muscle-specific transcriptional factors. Immature skeletal muscle cells consist of myoblasts proliferating and fusing into myotubes with the nuclei being centrally located. The completed immature multinucleated skeletal muscle cells then move the nuclei under the plasma membrane to develop into mature skeletal muscle cells during embryonic development [25]. Most studies concerning skeletal muscle cells work with mature skeletal muscle cells or tissue [6, 23–25, 27–29].

Kim et al. [30] performed a thermotherapy treatment on participants to determine the effects of heat on thigh muscle. Their heat treatment consisted of applying heated garments to the thighs of the participants. They reported an increase of oxidative enzyme nitric oxide synthase and heat shock proteins after an 8-week chronic heat treatment on participants. While the increase of synthases and heat shock proteins indicated increased muscle strength and influenced muscle capillarization, no change in mitochondrial content occurred. Similar to the work of Kim et al. [30], most thermotherapy studies discovered a change of heat shock proteins as an effect from the heat stress [4, 6, 31]. The heat shock proteins are also part of the human body's natural defense by helping protein formation in the cells, degradation of irreparable peptides, translocation of organelles across membranes, and conduction of repairs within the cytoplasm [4]. Heat-stressed muscle cells have been reported to release heat shock proteins which, in turn, decrease oxidative stress resulting in increased sensitivity of the cells [4, 6, 31]. Geiger and Gupte [6] reported that heat shock proteins also promote defense against oxidative stress. The mitochondrion within the cells benefits directly from induced heat shock proteins dissipating ROS [6]. Although Kim et al. [30] did not notice an effect on the mitochondria from their heat treatments, other studies confirm that the mitochondria are indeed affected by heat shock proteins' actions even though those studies only performed chronic heat treatments rather than acute treatments [4, 6, 31]. Conversely, as the levels of ROS increase, so do the damaging effects of ROS to the mitochondria [6]. This study aimed to determine the cell viability and oxidative stress of two heat treatments on healthy (HSMM) and T2D (D-HSMM) human skeletal muscle myoblast cell lines. Measurement of both the cell viability and oxidative stress of heat treatments on skeletal muscle cells may provide more knowledge pertaining directly to ROS levels than measuring the heat shock proteins produced during heat treatments.

## 2. Materials and Methods

### 2.1. Healthy and Type 2 Diabetes Human Skeletal Muscle Myoblast Cell Culture

**Figure 1:**
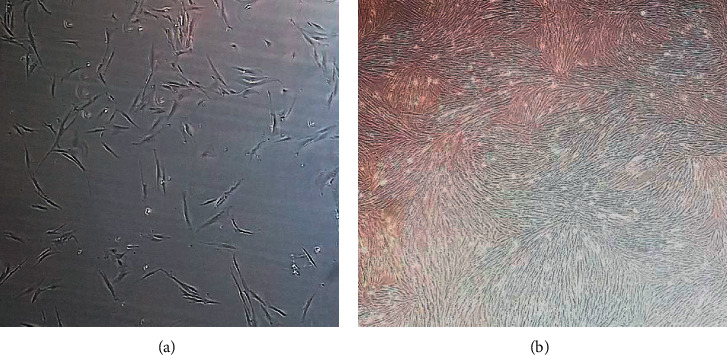
Depictions of cell confluency: (a) beginning of cell culture growth (<20% confluency) to (b) readiness for passaging (85–95% confluency).

HSMM and D-HSMM cells were purchased from Lonza Inc., Walkersville, MD, and incubated at 37°C and 5% CO_2_ in Skeletal Muscle Growth Media-2 (SkGM-2) (Lonza Inc.). To grow the cells, they were thawed in a 37°C water bath, transferred into a preincubated (45 minutes minimum) T-25 flask containing 5 mL of SkGM-2, and incubated in a 37°C and 5% CO_2_ incubator (VWR, Radnor, PA). When the media changed from bright pink to pale yellow (every 2–4 days depending on the culture), the media was removed and replaced with 5 mL of fresh pink culture media until the cultures reached 85–95% confluency. Confluency was assessed by cell coverage of the flask's bottom with 85–95% cells which appear more as a tissue rather than individual cells (Figure 1). The SkGM-2 was made with the SkGM-2 SingleQuots Kit, with Epidermal Growth Factor (hEGF), dexamethansone, L-glutamine, Fetal Bovine Serum (FBS), and Gentamincin/Amphotericin-B (GA), added with Basal Medium and Penicillin-Streptomycin (1000 units/mL) (Thermo Fisher Scientific) and frozen at −20°C when not in use [32].

To passage the cells, the media were removed, then 5 mL Hanks Balanced Salt Solution (HBSS) (Thermo Fisher Scientific) was used to wash the cells and removed, and 2 mL of Trypsin-EDTA Solution (Trypsin) was added to the cells. After 6 min, 4 mL of Trypsin Inhibitor Solution (TNS) was added before all were transferred into a sterile 15 mL centrifuge tube. To recover any remaining cells, 2 mL HBSS was used to rinse the flask and added to the centrifuge tube. Following centrifugation (Beckman Coulter, Allegra 6R Centrifuge) at 1000 rpm for 5 min, the supernatant was removed, and the pellets were resuspended in SkGM-2 to a final volume of 1 mL. One hundred *µ*L of the resuspension was removed for counting the cells using a hemocytometer and Trypan Blue (Corning Cellgro, Virginia), live cells being white perfect circles and dead cells being blue disrupted circles. The following equation was used for determining cell viability:

(1) $$\begin{array}{c} \%\text{ Viable Cells}=\frac{\left(\#\text{ Live Cells}\right)}{\left(\text{Total }\#\text{ Cells}\right)}\times 100. \end{array}$$

The remaining cells were transferred into new sterile flasks, suspended in SkGM-2 (5 or 15 mL depending on the T-25 or T-75 flask) and incubated at 37°C and 5% CO_2_ or frozen in cryofreeze media (−80°C) for future use [32].

To cryopreserve cells, new media were prepared using a 0.2 microfilter, SkGM-2, DMSO, and FBS in a 7 : 1:2 ratio. The cells were then placed into 1-2 mL cryovials, transferred into liquid nitrogen, and stored in a −80°C freezer until further use [32].

### 2.2. Heat Treatments

Both HSMM and D-HSMM cell lines were heat stressed for chronic treatment or acute treatment based loosely on Hooper's hot-tub therapy design [33]. Control HSMM and D-HSMM cells were not heat stressed. For chronic heat treatment, cells were transferred from 37°C to 40°C for 30 minutes and returned to 37°C at the same time three days a week over a three-week period for a total of 9 days. For acute treatment, the cells were transferred from 37°C to 40°C for 30 minutes and returned to 37°C once before assessing cell viabilities and further oxidative stress assays. Oxidative stress assays were run 24 hours following the final heat transfer in both treatments.

### 2.3. Oxidative Stress Assays

The cell pellets were harvested 24 hours after the chronic and acute heat treatments on HSMM and D-HSMM for the following assays: malondialdehyde (MDA), superoxide dismutase (SOD), and catalase. Controls were prepared with only 37°C incubation and cell culture media applied to the HSMM and D-HSMM cells. A blank control containing all final reaction mixture components of the assay except the cell pellets was also run in each assay.

### 2.4. Malondialdehyde Assay

The cells were sonicated in 1 mL ice-cold physiological saline (0.9% NaCl dissolved in H_2_O) and centrifuged at 28000 rpm for 5 minutes at 4°C. The supernatant was removed, and total protein in the cellular supernatant was determined by using a Stanbio kit (Stanbio Laboratory, Boerne, TX) [34]. The remaining cellular supernatant was added to the final reaction mixture of 3 mL containing 1.5 mL of 10 mmol/L potassium phosphate buffer (pH 7.4), 0.5 mL of the cellular supernatant (after total protein determination), 0.5 mL of 30% trichloroacetic acid (TCA), and 0.5 mL of thiobarbituric acid (TBA) (0.53%). The final reaction mixture was heated to 80°C for 1 hr, cooled to room temperature, and centrifuged at 2700 rpm for 5 min, and the absorbance of the clear supernatant was measured at 532 nm (Spectronic Instruments, Spectronic 20D+) against a blank control [10, 11]. A standard curve was prepared using the MDA compound dissolved in 10 mmol/L potassium phosphate buffer. The result was expressed in nmol of MDA formed per mg protein per mL.

### 2.5. Superoxide Dismutase Assay

The cell pellets were lysed by sonication in 1 mL of sonication buffer and centrifuged at 1500 rpm for 5 minutes at 4°C, and total protein in the cellular supernatant was determined by using a Stanbio kit [34]. The sonication buffer consisted of the following: cold 20 mmol/L HEPES buffer (pH 7.2) containing 1 mmol/L EGTA, 210 mmol/L mannitol, and 70 mmol/L sucrose [35]. The final reaction mixture consisted of the following: 0.5 mL of 0.1 mol/L sodium phosphate buffer, 0.032 mL of 3.3 mmol/L ethylenediaminetetraacetic acid (EDTA), 0.06 mL of 8.1 mmol/L fresh pyrogallol (made at least 1 hour ahead of time in 0.1 mol/L sodium phosphate buffer), and the mL amount of cellular supernatant containing 10 *μ*g protein. The change in absorbance at 420 nm of the final reaction mixture was monitored for 2 minutes against a blank control containing everything but the cellular supernatant. The SOD activity in the final reaction mixture was related to half of maximal inhibition of pyrogallol autoxidation (as shown in the equations below with control = blank that contains none of the supernatant fluid) [11]. SOD activity was determined through the following equations [14]:

(2) $$\begin{array}{c} X\%\text{ inhibition}=\;\frac{\text{control sample}-\text{treatment sample}}{\text{control sample}},\\ 50\%=1\text{ unit of enzyme,}\\ Y=\frac{1}{50}X,\\ \text{Enzyme activity}=\;\frac{Y\text{ final}-Y\text{ initial}}{\text{time}}. \end{array}$$

### 2.6. Catalase Assay

The cell pellets were lysed with 1 mL 0.01 M phosphate buffer (pH 7.0–7.4) and then centrifuged at 700 rpm for 5 min. The supernatant was removed, and total protein in the cellular supernatant was determined by using a Stanbio kit [34]. The final reaction mixture of 3 mL had the following: 0.2 mL protein supernatant fluid (∼1 mg of protein), 0.4 mL of 0.2 M H_2_O_2_, 2 mL dichromate/acetic acid (made with 5% K_2_Cr_2_O_7_ in glacial acetic acid in a 1 : 3 ratio), and 0.4 mL of 0.01 M phosphate buffer. The final reaction mixture was then boiled for 10 minutes or until a color change occurred from blue to green and cooled to room temperature. The change in absorbance at 510 nm was monitored for 5 minutes against a blank control containing everything but the cellular supernatant [15]. A standard curve was prepared by using 97% liquid catalase with 0.01 M potassium phosphate buffer to make 10 mL solutions.

### 2.7. Statistical Analysis

Data were presented as mean ± standard error of the mean. Values were obtained from three independent experiments that were performed in triplicate. The results among different thermotherapy treatments were evaluated by one-way ANOVA (*p* ≤ 0.05). Post hoc analysis was performed using Tukey's multiple comparison test for significance level (*p* ≤ 0.05) [36, 37].

## 3. Results

### 3.1. Effects of Thermotherapy on HSMM and D-HSMM Cell Viability

**Figure 2:**
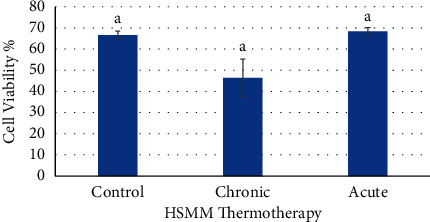
Effects of chronic and acute heat treatment on HSMM cell viability. Columns with the same letters are not significantly different from one another (*p* > 0.05).

The effects of chronic and acute heat treatments were investigated on cell viability in both healthy and diabetic human muscle myoblast cell lines (HSMM and D-HSMM).  Figure 2 shows no significant difference between the control and the chronic and acute treatments.

**Figure 3:**
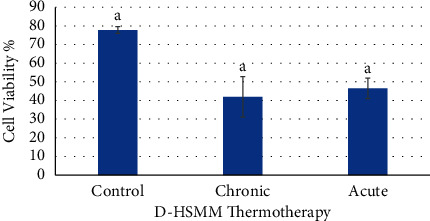
Effects of chronic and acute heat treatment on D-HSMM cell viability. Columns with the same letters are not significantly different from one another (*p* > 0.05).

Figure 3 shows cell viability in the D-HSMM cells following chronic and acute heat treatments. There was no significant difference between the chronic and acute treatment as compared to the control group. However, there was a decreasing trend promoted by the heat treatments.

### 3.2. Effects of Thermotherapy on Oxidative Stress

**Figure 4:**
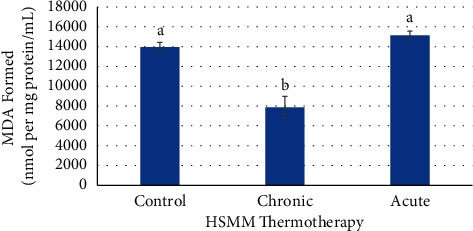
Effects of chronic and acute heat treatment on HSMM MDA formed. Columns with different letters are significantly different from one another (*p* ≤ 0.05). Columns with the same letters are not significantly different from one another (*p* > 0.05).

**Figure 5:**
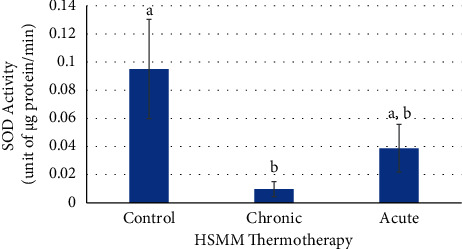
Effects of chronic and acute heat treatment on HSMM SOD activity. Columns with different letters are significantly different from one another (*p* ≤ 0.05). Columns with the same letters are not significantly different from one another (*p* > 0.05).

**Figure 6:**
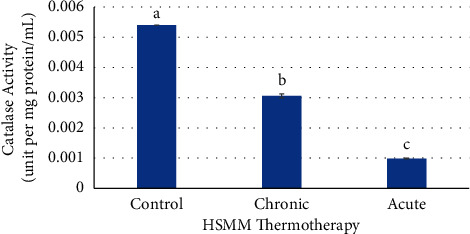
Effects of chronic and acute heat treatment on HSMM catalase activity. Columns with different letters are significantly different from one another (*p* ≤ 0.05). Columns with the same letters are not significantly different from one another (*p* > 0.05).

Figures 4–6 show the effects of chronic and acute thermotherapy on oxidative stress in HSMM cells. The control was not heat stressed. The chronic heat treatment had significantly decreased (*p* ≤ 0.05) MDA levels when compared to the control and acute heat treatments in Figure 4. The acute heat treatment had significantly increased (*p* ≤ 0.05) MDA levels compared to the chronic treatment. The chronic treatment had a significant decrease (*p* ≤ 0.05) of MDA concentration compared to the control. The acute heat treatment had no significant increase (*p* > 0.05) of MDA concentration when compared to the control.

The SOD activity in the chronic heat treatment was significantly decreased (*p* ≤ 0.05) compared to the control. The SOD activity in the acute heat treatment had no significant change (*p* > 0.05) when compared to either chronic heat treatment or the control (Figure 5).

Chronic treatment had a significant decrease (*p* ≤ 0.05) when compared to the control and a significant increase (*p* ≤ 0.05) in catalase activity when compared to the acute treatment. The acute heat treatment had a significant decrease (*p* ≤ 0.05) in catalase activity when compared to the control (Figure 6).

**Figure 7:**
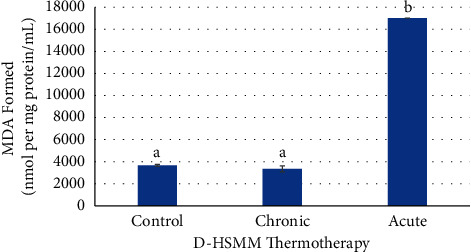
Effects of chronic and acute heat treatment on D-HSMM MDA formed. Columns with different letters are significantly different from one another (*p* ≤ 0.05). Columns with the same letters are not significantly different from one another (*p* > 0.05).

**Figure 8:**
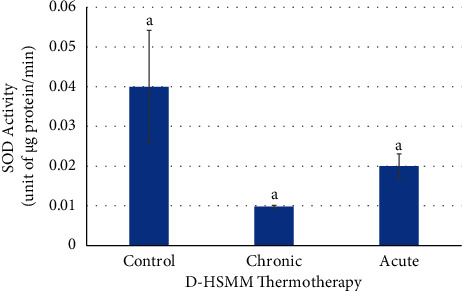
Effects of chronic and acute heat treatment on D-HSMM SOD activity. Columns with the same letters are not significantly different from one another (*p* > 0.05).

**Figure 9:**
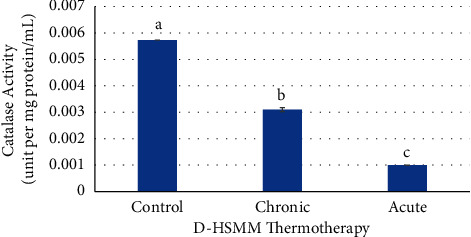
Effects of chronic and acute heat treatment on D-HSMM catalase activity. Columns with different letters are significantly different from one another (*p* ≤ 0.05). Columns with the same letters are not significantly different from one another (*p* > 0.05).

Oxidative stress of chronic and acute thermotherapy in D-HSMM cells is shown in Figures 7–9 . The control was not heat stressed. The chronic heat treatment had significantly decreased (*p* ≤ 0.05) MDA levels when compared to the acute heat treatment, but no significant change (*p* > 0.05) on MDA concentration when compared to the control. The acute heat treatment showed significantly increased (*p* ≤ 0.05) MDA levels over both the chronic heat treatment and the control (Figure 7).

Both chronic and acute heat treatments showed no significant changes (*p* > 0.05) in SOD activity compared to the control (Figure 8). The observed no significant changes among the groups is due to the >100% coefficient of variation for the control.

Chronic treatment had a significant increase (*p* ≤ 0.05) in catalase activity when compared to the acute treatment but had a significant decrease (*p* ≤ 0.05) when compared to the control. The acute heat treatment had a significant decrease (*p* ≤ 0.05) in catalase activity when compared to the control (Figure 9).

## 4. Discussion

Due to the ever-increasing prevalence of type 2 diabetes (T2D) in the world, especially in the elderly population, a regimen of diet, drugs, and exercise has become the usual advice of physicians [1]. Exercise has been highly recommended and cited to be an effective natural treatment of T2D [38]. As a potential form of exercise mimetic for T2D, thermotherapy mimics the increase in body temperature that is produced by exercise [27, 30, 33]. Heat stress treatments using either water or air have demonstrated beneficial effects such as increased heat shock proteins, reduction of oxidative stress, and increased insulin sensitivity [39, 40]. Heat stressing the skeletal muscle cells in this study's thermotherapy premise resembles the muscles themselves generating an increased heated environment during exercise [27, 30, 33]. This study assessed the effectiveness of two heat stress treatments on HSMM and D-HSMM cells. Racinais et al. [41] reported that passive heat acclimation, similar to the heat treatments in this study, improved human's skeletal muscle function. However, this study's thermotherapy data indicated no beneficial promotion of cell viability in HSMM and D-HSMM cells. The benefits of thermotherapy on these cell lines may become more evident with longer exposures of heat and provide a better assessment of the treatments. This focus is currently under investigation in our lab.

As oxidative stress has been linked to be a cause of multiple chronic illnesses and aging, lipids have been determined to be one of the major components of oxidative stress. Lipid oxidation produces highly toxic secondary products such as the well-studied malondialdehyde (MDA) [42]. ROS free radicals and H_2_O_2_ were not directly assessed in this study. Instead, MDA as a secondary by-product of ROS interactions was measured. Data from this study showed an increase in MDA concentrations while the biological defense markers superoxide dismutase (SOD) and catalase were reduced in both HSMM and D-HSMM cells.

MDA was significantly increased in the acute heat treatment compared to the control and the chronic treatment in both the HSMM and D-HSMM cell lines and may indicate a presence of intramyocellular lipid accumulation in these skeletal muscle cells [29]. The availability of the lipids may have allowed more ROS reactions resulting in the increased production of MDA. The heat stress may also have provided an energy surplus to produce lipid peroxides from the lipids and fatty acids from the mitochondria resulting in more ROS [43, 44].

Meilhac et al. [45] researched the addition of lipids to different cell lines and demonstrated an increase of catalase activity in rabbit femoral arterial smooth muscle cells in response to lipids' addition before the induction of H_2_O_2_. They suggested that other biological defense markers such as SOD activity may have been activated simultaneously as catalase. Because the chronic heat treatment lasted 3 weeks, there may have been enough time for the biological defenses to activate and remove the excess lipids in the intramyocellular region. With additional time, the cells in the chronic treatment may have returned to a new equilibrium state where the ROS only reacted with the bilayer lipid membranes instead of excess lipids resulting in less MDA production [7, 8, 16, 17, 29, 42, 46].

SOD provides protection from superoxide radicals by forming hydrogen peroxide (H_2_O_2_) from superoxide anions (O_2_^−^). These radicals must be kept in check because they can produce additional reactive species. Powers et al. [47] noted several studies reporting either an increase or decrease of SOD activity after endurance exercises. Since they found more studies supporting an increase of SOD activity after an exercise regimen, they suggested more intense exercise routines caused an increase of SOD. SOD demonstrated low activity compared to the control group in both HSMM and D-HSMM cells for the chronic and acute heat treatments. The low activity of SOD may indicate that heat stressing the cells was not at enough intensity in this study. Another explanation could be there was very low levels of O_2_^−^ to convert to the less reactive H_2_O_2_ molecules within the cell [17]. The ROS may have reacted primarily with the cells' bilayer lipid membrane to produce MDA, leaving very few remnants of O_2_^−^ for SOD to be active [7, 8, 17, 46].

Catalase is an antioxidant enzyme that catalyzes the breakdown of H_2_O_2_ into H_2_O and O_2_ and is widely distributed within the cells. Similar to SOD activity, Powers et al. [47] noted no consensus across multiple studies of catalase activity increasing or decreasing based on an exercise regimen. The catalase activity was consistent throughout both chronic and acute heat treatments for both HSMM and D-HSMM cell lines in this study. The control cells in both cell lines maintained the most catalase activity when compared to the heat treatments suggesting a defective antioxidant defense system. Unuofin and Lebelo [46] reported that a defective antioxidant defense system can occur as ROS increases and SOD and catalase activities decrease.

Catalase demonstrated very low activity in the chronic and acute heat treatments compared to the control heat treatment in both HSMM and D-HSMM cell lines. As catalase follows SOD by transforming H_2_O_2_ molecules into H_2_O and O_2_ [22], low activities of SOD and catalase in tandem suggest decreased concentrations of O_2_^−^ and H_2_O_2_ molecules in the ROS activities [17, 22].

This study found that the chronic heat treatment consistently had a significant increase in catalase activity compared to the acute treatment. The chronic treatment's extended time span may indicate a new equilibrium was already in place or in the process of being established in response to the increased MDA values from the first heat stress. As MDA can be an indicator of excess lipid presence, the chronic treatment may have had enough time to activate catalase in response to the lipid peroxides regardless of the presence of H_2_O_2_ molecules [45]. This new equilibrium would result in increased catalase activity and decreased MDA levels.

Rather than lowering the ROS activity of MDA production compared to the biological defenses of SOD and catalase [17], heat stressing the HSMM and D-HSMM cells seems to have increased oxidative stress after 24 hr. Heat stressing the cells may have input heat energy into the ROS interactions with the cell membranes causing a chain reaction of increased ROS-MDA production [8, 16, 46]. The chain reaction may have caused dysfunctional mitochondria due to ROS's reactivity following heat stress resulting in an increase of oxidative stress in the cells [3, 6, 28]. Yokota et al. [48] found that increased oxidative stress over a period of eight weeks may have caused mitochondrial dysfunction in their T2D mice. This study's chronic heat treatment may have experienced a similar result in mitochondrial dysfunction from an increase of oxidative stress.

Although both healthy and T2D cells experienced oxidative stress in the heat treatments, increased dysfunctional mitochondria may cause the diabetic cells to have an increased susceptibility to insulin resistance [44, 48]. Future research may focus on T2D cells' mitochondria or insulin resistance in response to the heat stressors. As this study intentionally only looked at skeletal muscle cells, further research of thermotherapy's oxidative effects may focus on skeletal muscle fibers.

## 5. Conclusions

In conclusion, thermotherapy had no significant effect on the cell viability of HSMM and D-HSMM cells. Thermotherapy was not effective in curtailing the indices of oxidative stress in both cells, especially the acute treatment in the D-HSMM cells.

## Data Availability

The datasets used and/or analysed during the current study are available from the corresponding author on reasonable request.
